# 

**Published:** 1923-12

**Authors:** 


### HOSPITAL and HEALTH REVIEW

*Established 1886* | *No.* 1869. *Vol. LXXII.*

*New Series.* Vol. II No. 27 *December,* 1923. Price Sixpence.

CONTENTS.

| | PAGE |
|---|---|
| Modern "Bills of Mortality" | 419 |
| "Abolishing" the Slums | 420 |
| Notes and Comments:— The Ministry Again in Danger—Beginning at the Beginning—Effluvia and Efflorescence—International Health—One of the Primitives—Asmodeus at Large—Popular Health Literature—The Healing Sun—Feeding Weakly School Children—Unfit for Babies—Effect of Smoking—Betting and Ballots—The Development of Warts—"Colour-Hearing" and Heredity—Restoring Faded Records—Microscoping the Living Eye | 421 |
| The Prince in His Principality: A Hospital Tour | 425 |
| Hospitals and Country Annexes | 426 |
| Health of the School Child | 427 |
| The London Hospitals: Analysis of the Statistical Report | 428 |
| The Public Health: Interviews with Local Authorities—Essex | 429 |
| Great Northern Festival Dinner | 431 |
| London and Liverpool Hospitals | 431 |
| The Panel Doctors' Decision | 432 |
| Health Exhibition at Leeds | 432 |
| The Outlawry of Disease | 433 |
| "Our Ostriches" | 433 |
| Clothing and Health | 434 |
| League of Remembrance | 435 |
| Hospital Progress and Finance | 436 |
| Reviews of Books | 438 |
| Correspondence | 441 |
| Echoes of the Month | 443 |
| Hospital and Health News | 445 |
| Recent Appointments | 446 |
| Hospital Needs and Hospital Appeals | 447 |

